# Increasing involvement of *CAPN1* variants in spastic ataxias and phenotype-genotype correlations

**DOI:** 10.1007/s10048-020-00633-2

**Published:** 2021-01-23

**Authors:** Jean-Loup Méreaux, Cristina Firanescu, Giulia Coarelli, Malin Kvarnung, Rita Rodrigues, Elena Pegoraro, Meriem Tazir, Frédéric Taithe, Rémi Valter, Vincent Huin, Kristina Lidström, Guillaume Banneau, Sara Morais, Livia Parodi, Marie Coutelier, Mélanie Papin, Per Svenningsson, Jean-Philippe Azulay, Isabel Alonso, Daniel Nilsson, Alexis Brice, Eric Le Guern, Rayomand Press, Giovanni Vazza, José Leal Loureiro, Cyril Goizet, Alexandra Durr, Martin Paucar, Giovanni Stevanin

**Affiliations:** 1 Sorbonne Université, Institut du Cerveau - Paris Brain Institute - ICM, Inserm, CNRS, APHP, Hôpital de la Pitié Salpêtrière, DMU Neuroscience 6, Paris, France; 2 Rouen University Hospital, Rouen, France; 3 Paris Sciences et Lettres University, EPHE, Paris, France; 4 Department of Neurology, Karolinska University Hospital, Stockholm, Sweden; 5 APHP, National Reference Center for Rare Diseases ‘Neurogenetic’, Department of Genetics, Pitié-Salpêtrière University Hospital, Paris, France; 6 Department of Clinical Genetics, Karolinska University Hospital, Stockholm, Sweden; 7 Department of Molecular Medicine and Surgery, Center for Molecular Medicine, Karolinska Institutet, Stockholm, Sweden; 8 Neurology Department, Centro Hospitalar Entre Douro e Vouga, Santa Maria da Feira, Portugal; 9 Department of Neurosciences DNS, ERN Neuromuscular Centre, University of Padua, Padua, Italy; 10 Laboratoire de Recherche en Neurosciences, Université d’Alger 1, Service de Neurologie, CHU Mustapha, Place du 1er Mai, 16000 Alger, Algeria; 11 CHU de Clermont-Ferrand, 63000 Clermont-Ferrand, France; 12 APHP, Department of Genetics, Pitié-Salpêtrière University Hospital, Paris, France; 13 UnIGENe, IBMC - Institute for Molecular and Cell Biology, i3S - Instituto de Investigação e Inovação em Saúde, Universidade do Porto, Porto, Portugal; 14 Department of Biology, University of Padua, Padua, Italy; 15 Department of Clinical Neuroscience, Karolinska Institutet, Stockholm, Sweden; 16 Département de Neurologie et Pathologie du Mouvement, Pôle Neurosciences Cliniques, INT-CNRS/AMU Aix-Marseille, Marseille, France; 17 Present Address: Genetyca-ICM, Porto, Portugal; 18 National Reference Center for Rare Diseases ‘Neurogenetic’, Department of Medical Genetics, Pellegrin Hospital, Bordeaux University Hospital, Bordeaux, France; 19 Rare Diseases Laboratory: Genetics and Metabolism (MRGM), INSERM U1211, Bordeaux University, Bordeaux, France; 20 Institut du Cerveau (ICM), Pitié-Salpêtrière Hospital, CS21414 - 47 bd de l’Hôpital, 75646 Paris, France

**Keywords:** *CAPN1*, Spastic ataxia, Neurodegeneration, Spastic paraplegia, Cerebellar ataxia

## Abstract

Spastic ataxias are rare neurogenetic disorders involving spinocerebellar and pyramidal tracts. Many genes are involved. Among them, *CAPN1*, when mutated, is responsible for a complex inherited form of spastic paraplegia (SPG76). We report the largest published series of 21 novel patients with nine new *CAPN1* disease-causing variants and their clinical characteristics from two European university hospitals (Paris and Stockholm). After a formal clinical examination, causative variants were identified by next-generation sequencing and confirmed by Sanger sequencing. *CAPN1* variants are a rare cause (~ 1.4%) of young-adult-onset spastic ataxia; however, together with all published cases, they allowed us to better describe the clinical and genetic spectra of this form. Truncating variants are the most frequent, and missense variants lead to earlier age at onset in favor of an additional deleterious effect. Cerebellar ataxia with cerebellar atrophy, dysarthria and lower limb weakness are often associated with spasticity. We also suggest that cognitive impairment and depression should be assessed specifically in the follow-up of SPG76 cases.

**Supplementary Information:**

The online version contains supplementary material available at 10.1007/s10048-020-00633-2.

## Introduction

Spastic ataxias are rare neurodegenerative genetic disorders involving spinocerebellar and pyramidal tracts. They belong to the clinical spectrum of hereditary spastic paraplegias (HSPs) and hereditary cerebellar ataxias (HCAs). Variants in more than a hundred genes have been implicated in their etiology [1, 2]. All classical transmission modes have been described.

*CAPN1* variants were initially associated with an autosomal recessive (AR) form of complex HSP (SPG76) or spastic ataxia in 2016 [3, 4]. Since then, an increasing number of patients have been described (*n* > 50). *CAPN1* encodes the calpain-1 protein, a calcium-activated intracellular proteinase, with numerous biological roles, including in synaptic plasticity and neuroprotection [5].

Gene panel screening and exome or genome sequencing in two European university hospitals (Paris and Stockholm) allowed us to diagnose 21 novel cases carrying 13 *CAPN1* causative variants, the largest series reported so far. We report here the clinical and genetic characteristics of this cohort, including nine novel variants. Furthermore, we explored genotype-phenotype correlations based on our findings and a review of all previously published cases.

## Materials and methods

Patients were clinically examined according to described clinical criteria (https://spatax.wordpress.com/downloads/). Disease severity was measured using the SPATAX disability scale (0: no functional handicap; 1: no functional handicap but signs at examination; 2: mild, able to run, walking unlimited; 3: moderate, unable to run, limited walking without aid; 4: severe, walking with one stick; 5: walking with two sticks; 6: unable to walk, requiring wheelchair; 7: confined to bed). The study was approved by the Stockholm and Paris-Necker Ethics Committees. All patients signed informed consent for genetic analyses, which were conducted in accordance with the relevant national ethical rules. Their DNA collected from blood cells was used for genome sequencing (six probands), exome sequencing (two probands), or targeted gene panel sequencing (five probands with four among 182 probands previously excluded for pathogenic variants in 65 HSP genes [6]). Standard procedures were used for next-generation sequencing, sequence alignment and filtering, as described elsewhere [6, 7]. All variants were confirmed by Sanger sequencing, and their co-segregation with the disease was also verified in other family members when DNA was available. Novel variants have been submitted to the ClinVar database.

The literature review was done from all *CAPN1*-mutated cases reported in PubMed or ClinVar before October 2020. The search was done in PubMed with ‘CAPN1’ as the search term, and the papers reporting a description of cases harboring variants in *CAPN1* were kept for the review.

Mean and standard deviation were used to describe continuous quantitative variables, and median and interquartile range (iqr) were used for discrete quantitative variables. Student’s *t* test was used to compare means of onset age, disease duration and handicap that followed a normal distribution, as assessed visually and by Shapiro-Wilk test. Chi2 test was used to compare distributions of clinical features. A *p* value < 0.05 was considered as statistically significant.

## Results

### Case series

**Fig. 1:**
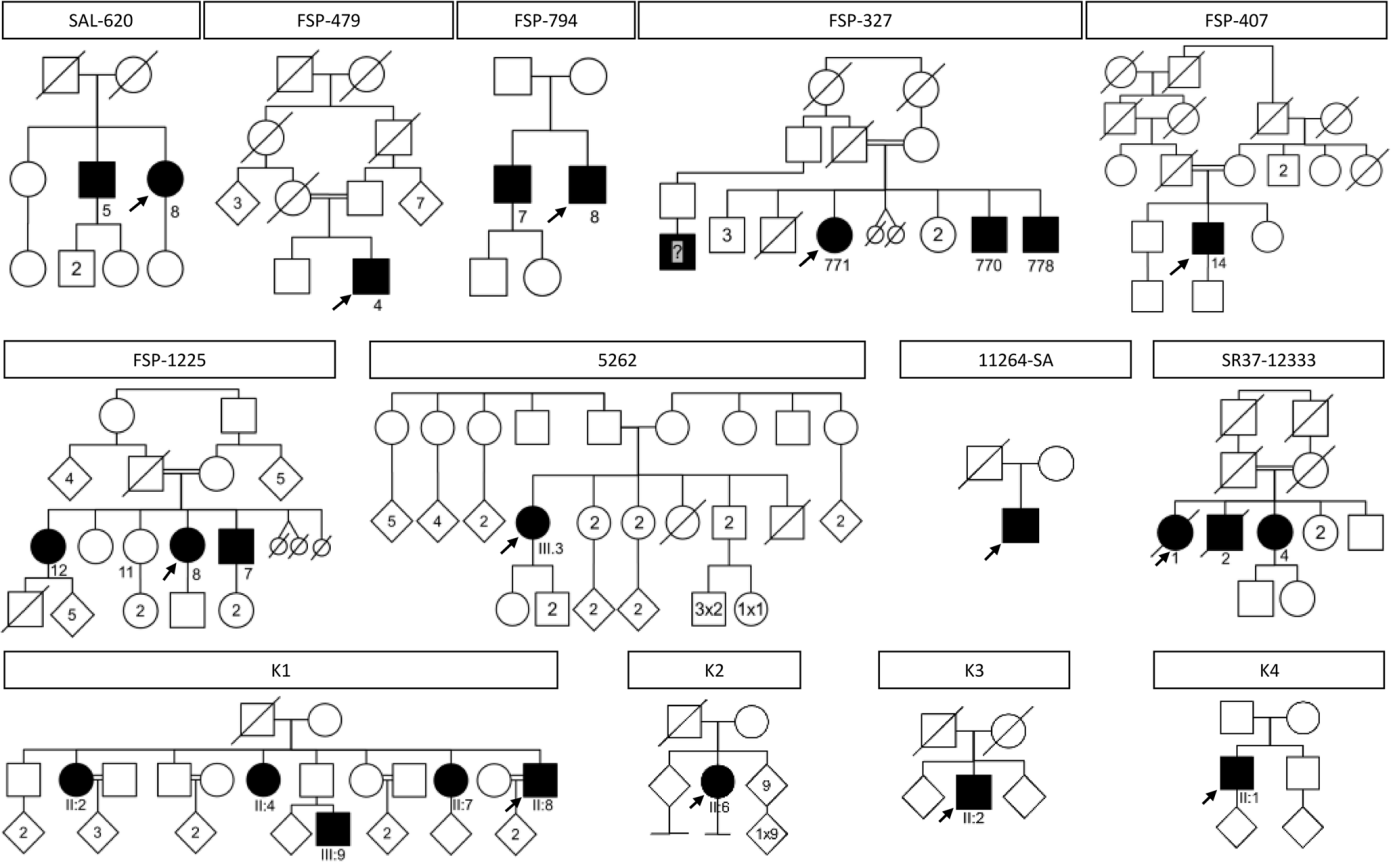
Pedigrees of *CAPN1* families. Affected filled in black. Squares for men, circles for women, lozenges for men or women, small circles if stillborn. Numbers in squares and circles denote the number of people with similar characteristics to simplify the figure. Below the symbols are indicated the anonymous DNA sample identifier. Arrows show the probands

We diagnosed a series of 21 cases of spastic ataxia with *CAPN1* causative variants from 13 new families (Fig. 1). Clinical and genetic data are summarized in Table 1 and full clinical data are shown in Supplementary Table 1.

**Table 1:** *CAPN1*-mutated cases with clinical features (proband in italics; F: female; M: male; +: present, −: absent; N: normal; A: abnormal; na: not available; SPATAX disability score for severity; variants for CAPN1 transcript NM_001198868 with new reported in italics)

Family	FSP-479	FSP-794		FSP-327			K4	K3	11264-SA	FSP-407	SR37-12333		Family	SAL-620		5262	FSP-1225				K1		K2
Individual	*4*	7	*8*	770	*771*	778	*II:1*	*II:2*		*14*	*1*	4	Individual	5	*8*	*1*	7	*8*	12		*II:8*	II:7	*II:6*
Gender	M	M	M	M	F	M	M	M	M	M	F	F	Gender	M	F	F	M	F	F		M	F	F
Origin	France	Portugal		Algeria			Czech Republic	Sweden	Italy	Portugal	Portugal		Origin	France		France	Egypt				Iraq (Kurdish)		Iraq
Variant 1	c.254G>A (p.W85*)	c.618_619del (p.G208Qfs*7)		*c.623G>A (p.G208D)*			c.1005G>A (p.W335*)	c.1129_1133del (p.R377Efs*25)	c.1153C>T (p.R385*)	c.1176G>A (p.W392*)	c.1176G>A (p.W392*)		Variant 1	c.1176G>A (p.W392*)		c.1605+5G>A (p.E523Kfs*28) [SPLICE]	c.1697dup (p.L566Ffs*7)				c.1969G>T (p.E657*)		c.1969G>T (p.E657*)
Variant 2		*c.1341G>C (p.?) [SPLICE]*					c.1605+5G>A (p.E523Kfs*28)[SPLICE]	*c.1165+1G>A (p.?)[SPLICE]*					Variant 2	c.1418_1419del (p.R473Lfs*53)									
Consanguinity	+	−		+			−	−	−	+	+		Consanguinity	−		−	+				−		−
Age at onset (years)	16	34	31	19	24	6	30	22	14	15	28	30	Age at onset (years)	28	38	42	18	23		36	20	28	24
Age at exam (years)	24	38	37	25	32	21	50	31	43	27	60	55	Age at exam (years)	58	48	43	36	35	41	45	44	48	54
Severity	2	1	1	3	4	2	3	3	4	3	5	4	Severity	5	3	3	4	3	4	5	3	5	5
UL spasticity/hyperreflexia	+/+	na/na	na/+	+/+	+/+	+/+	−/−	−/−	+/+	−/+	+/+	+/+	UL spasticity/hyperreflexia	+/+	+/+	+/+	na/na	−/+	−/+	−/na	−/−	−/+	−/+
LL weakness	+	−	−	+	+	+	+	−	+	−	+	+	LL weakness	+	+	+	na	+	+	+	+	+	+
Dysarthria	+	−	−	+	+	+	−	−	+	−	−	+	Dysarthria	+	+	−	na	−	+	+	−	+	+
Dysphagia	−	−	−	−	−	−	−	−	−	−	−	−	Dysphagia	+	+	−	na	−	−	na	−	+	+
Cerebellar ataxia	+	+	−	+	+	+	−	−	+	−	−	−	Cerebellar ataxia	+	+	+	na	−	+	na	−	−	−
Sensory deficit	−	+	+	−	−	−	−	−	+	−	+	+	Sensory deficit	+	+	−	na	−	−	na	−	−	−
Skeletal anomalies	+	+	+	−	−	−	−	−	−	−	−	−	Skeletal anomalies	+	+	−	na	+	+	na	−	−	−
Psychiatric features	−	na	na	−	−	−	−	+	−	+	−	−	Psychiatric features	+	+	−	na	+	+	na	−	+	+
Brain/spine MRI	N/na	na/na	na/na	na/na	na/na	A/na	N/N	N/N	N/na	A/N	N/na	N/na	Brain/spine MRI	A/na	N/N	A/N	na/na	N/A		na/na	N/N	N/N	N/N

Thirteen different disease-causing variants were found, and nine of these are reported here for the first time: c.254G>A (p.Trp85*), c.623G>A (p.Gly208Asp), c.1697dup (p.Leu566Phefs*7), and c.1969G>T (p.Glu657*) at the homozygous state, and c.1005G>A (p.Trp335*), c.1129_1133del (p.Arg377Glufs*25), c.1165+1G>A (p.?), c.1341G>C (splice site variant), and c.1418_1419del (p.Arg473Leufs*53) at the compound heterozygous state. These novel variants were not reported in gnomAD [8]. In total, half of the cases were from consanguineous unions, explaining a high homozygosity rate of 71% (15 cases). All our variants were truncating except one (8%). The only non-truncating damaging missense variant, c.623G>A (p.Gly208Asp), affected a highly conserved residue and was predicted to alter the protein function by all commonly used prediction scores (CADD, MutationTaster2, PolyPhen-2, and SIFT) [9–12]. The c.1341G>C variant was considered as truncating as it was highly predicted to affect the splicing by deletion of the splicing donor site at the end of exon 11 (prediction with MaxEntScan − 100.0%, NNSPLICE − 94.6% and SSF − 16.2%) [13–15]. The study of mRNA from white blood cells of the proband identified multiple abnormal transcripts compared with controls, one of which could be sequenced and shown to result from partial retention of the following intron, leading to a frame shift and a premature stop codon: p.Pro449Argfs*17.

The patients originated from various geographical locations: 11 from Western Europe, six from North Africa, one from Eastern Europe and three from Iraq. The sex ratio was 1.33 (*p* > 0.05). Mean age at onset of the disease was 25.1 ± 8.8 years, and mean age at examination was 41.0 ± 11.4 years. Disease severity using the SPATAX disability scale showed variable levels of handicap, with a median score of 3 (iqr 3–4) after a mean disease duration of 16.2 ± 9.7 years. Most of the patients had walking difficulties (17/21) with two at score 1, two at 2, seven at 3, five at 4 and five at 5. Lower limb spasticity, extensor plantar reflex and lower limb hyperreflexia were present in all patients whereas upper limb spasticity was found in 10 of the 18 cases with available data (56%). Lower limb weakness was present in 16/20 cases (80%). In 60% of cases (12/20), dysarthria was noted and was described as spastic (*n* = 5), bulbar (*n* = 3) or cerebellar (*n* = 4). Cerebellar ataxia was observed in 10/19 cases (53%). Other frequent features were sphincter dysfunction (primarily bladder) in 10/18 (56%), sensory deficit (always in deep modality and also superficial in one case) in 7/19 (37%), saccadic pursuit in 3/18 (17%) and dysphagia in 4/19 (21%). A single case among 19 patients with available examination (5%; patient FSP-1225-8) had extrapyramidal signs with the development of orofacial dystonia and hypokinesia during the follow-up, without any exposure to neuroleptics. Three affected relatives from family FSP-1225 presented an early bilateral cataract starting in their forties, which was also present in individual 11 who had no spastic ataxia. No eye fundus anomalies were detected. Skeletal abnormalities were reported in 6/19 cases (32%) as scoliosis, pes cavus, hollow foot, C2–3 fusion with supernumerary hemivertebra and unspecified dental abnormalities. Interestingly, a cognitive impairment was observed in two sisters (family SR37-12333, 2/19 or 11%) after three decades of disease evolution. In addition, 7/17 patients (41%) had psychiatric manifestations with mood disorders that manifested as depression, post-traumatic stress disorder or bipolar disorder. Epilepsy was not reported. Brain MRI was abnormal in 4/15 cases (27%) with cerebellar atrophy, clinically observed as ataxia in three of them. Spine MRI showed upper spine atrophy in 1/9 cases (11%). Neuropathy was confirmed by electroneuromyography (ENMG) in a single case (9%; patient FSP-327-771).

The clinical variability was evident between family members and between some affected cases sharing the same causative variant in different families. For example, the age at onset ranged from 18 to 36 years in family FSP-1225 (patients homozygous for c.1697dup) and it ranged from 15 to 30 years for the recurrent c.1176G>A variant in the two families carrying it at the homozygous state. Genotype to phenotype correlations could be found with neither the position nor the consequence of the variant in our series of 21 patients.

A prospective follow-up is given in Table 1 with two clinical examinations for patient FSP-1225-8 at 35 and 41 years old. It showed that the evolution of the disease course was progressive with a pure spastic paraparesis at onset complicated over time by cerebellar ataxia, orofacial dystonia and increased disability.

## Discussion

We report 13 *CAPN1* variants in 21 patients from 13 families, nine being novel causative variants. While some families were found through exome, genome or targeted panel sequencing in large cohorts of unselected patients with various phenotypes, four were identified in a series of 182 HSP patients screened for pathogenic variants in known HSP genes [6], allowing us to estimate the relative frequency of *CAPN1* variants in HSP patients. These 182 cases were from a series of 292 cases, 37.7% of whom were found to have mutations in known HSP genes (unpublished data), suggesting that *CAPN1* variants account for 1.4% of our European continental HSP patients. This is higher than in a *CAPN1* screening of 107 AR or sporadic HSP cases and 54 HCA Chinese patients, where a frequency of 0.6% was reported [16], but is in line with the frequency of 1/47 (2.1%) paediatric-onset HSP patients [17]. Due to the recent establishment of *CAPN1* as a disease gene, more cases could well be found in undiagnosed patients, particularly as the diagnosis process is evolving from targeted analysis of a few known disease genes to high-throughput analyses of the entire genome.

### Literature review

**Fig. 2:**
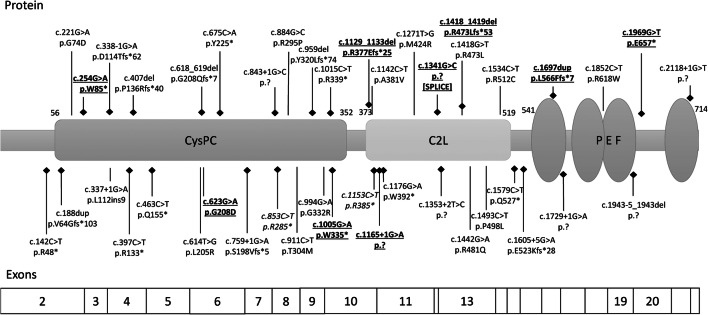
Calpain-1 with its 3 functional domains and all reported pathogenic variants (new in bold; truncating variants localized with a lozenge whereas a simple line for non-truncating variants. Below the protein are the corresponding *CAPN1* coding exons)

There are 34 previously published pathogenic *CAPN1* variants in 37 families or 62 cases in PubMed or ClinVar (Supplementary Table 2) [3, 4, 16, 18–33]. One of these variants was reported in ClinVar without clinical data (c.1153C>T) [VCV000489122.2], and we confirmed this variant as disease-causing in one of our cases. Consanguinity is frequent, accounting for 63% of all known families. Our new cases slightly increased the sex ratio to 0.76 from the previous ratio of 0.61, but the difference remains non-significant. Nevertheless, females are more frequently affected by complex forms (*p* < 0.01) as already reported [19]. All known variants are distributed throughout the whole gene (Fig. 2). Most variants were private, affecting a single family except for the most frequent, c.1176G>A, present in seven families, c.1605+5G>A found in four families, c.188dup and c.759+1G>A both identified in three families, and c.618_619del, c.853C>T, c.1142C>T, c.1153C>T, c.1493C>T, c.1534C>T and c.1969G>T, each detected in two families. A common ancestor could be hypothesized for the most frequent variant, c.1176G>A, since some mutated cases shared common origins, such as Brazil or Portugal (the others were from Turkey and France). In contrast, there was no known common origin for the four families carrying the c.1605+5G>A variant, which affects a CG doublet, known as a potential mutational hotspot and thus making a founder effect less likely. The various types of variants occurring in this gene are nonsense, missense, frameshift, and splice site variants. Truncating variants are frequent and represent 29 (67%) of the 43 *CAPN1* causative variants now known with our supplementary series or also 40 (80%) of the 50 known families. The number of truncating variants could be slightly overestimated due to predicted splicing variants that may not have truncating consequences, as is the case for c.337+1G>A (p.Leu112ins9) [4]. Indeed, the reported splicing variants have rarely been functionally studied for their consequences (*n* = 7/11 without mRNA analysis).

**Fig. 3:**
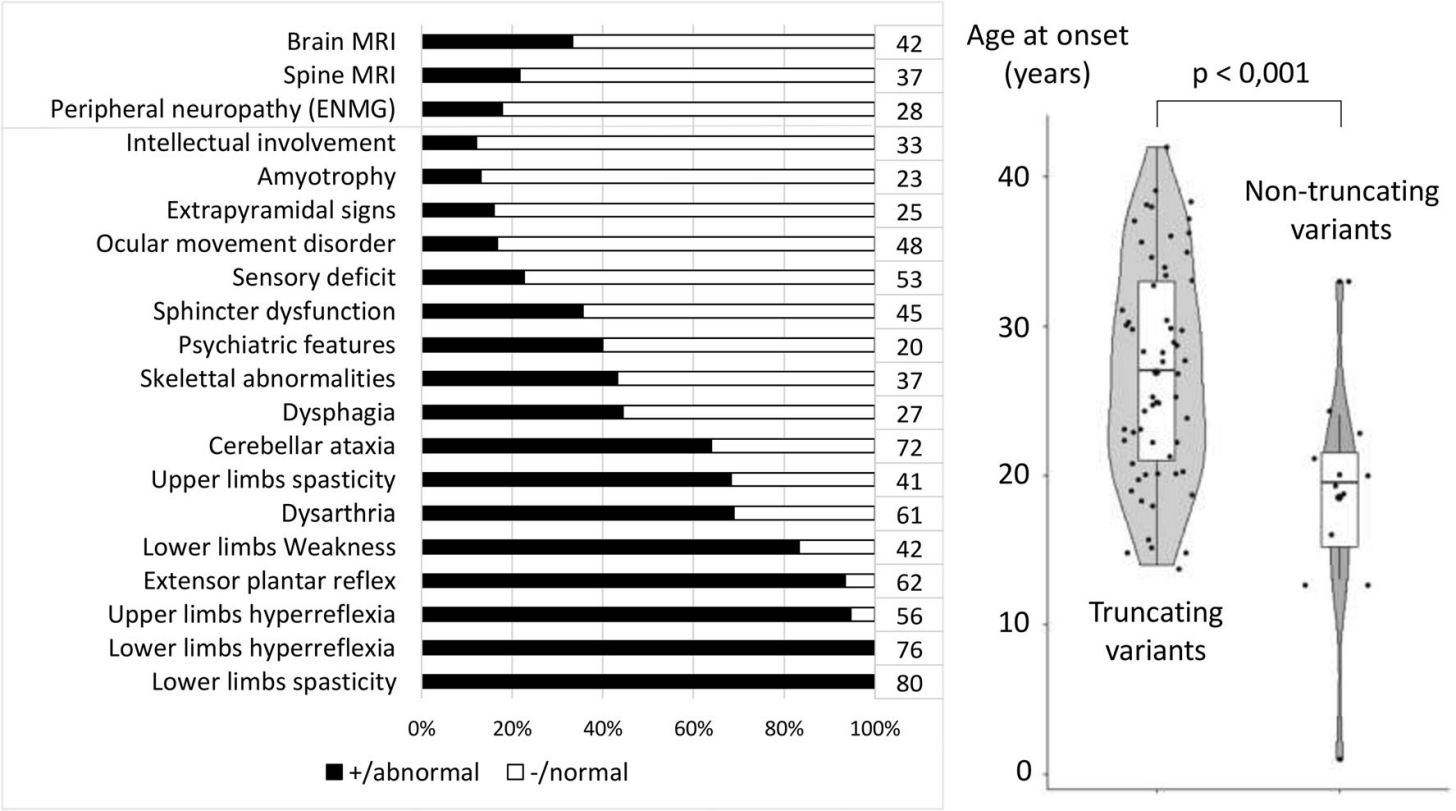
Clinical summary of the 83 *CAPN1*-mutated cases reported in the literature, including our 21 cases (number at the end of each line indicates the number of cases with available data)

As reported recently, the frequency of truncating variants did not differ between pure and complex forms [19] but, interestingly, we found that they are associated with a later onset than non-truncating missense variants (mean of 26.9 ± 7.2 vs 17.5 ± 8.1 years, *p* < 0.001, Fig. 3). We therefore hypothesize that the pathogenic missense variants in *CAPN1* could cause additional negative effects, although their smaller number (*n* = 16 cases) makes conclusions difficult especially as the handicap does not differ after the same disease duration (*p* = 0.3) of 12.1 ± 9.0 years for the missense variants and 14.8 ± 9.0 years for the truncating variants. The case with the earliest onset, at 1 year, supports this hypothesis of an increased severity of missense variants; but this patient was reported to carry three missense variants (c.221G>A (p.Gly74Asp) + c.911C>T (p.Thr304Met) + c.1418G>T (p.Arg473Leu)), all predicted to affect protein structure [32]. The second earliest cases, two patients with onset at 13 years, were also caused by missense variants [26, 30]. In truncating variants, the earliest onset was 14 years in c.1153C>T (p.Arg385*) in one of our families (11264-SA).

Pure HSPs are estimated at 36% (Clopper-Pearson confidence interval 0.95, 26–47%) of all known *CAPN1* cases, but missing reported clinical data could lead to this being overestimated and the progression of the neurodegeneration could complicate the initial pure clinical presentation in some patients. The distribution of associated clinical features of *CAPN1* variants from all known cases is summarized in Fig. 3 to show the global clinical signature of *CAPN1*-mutated cases. Lower limb spasticity is a constant feature, and spasticity often extends to the upper limbs (68% or 28/41 cases with available data). Thereafter, cerebellar ataxia, dysarthria and lower limb weakness are the most frequent signs. Dysarthria is more frequent in AR than in autosomal dominant (AD) forms of HSP. It is found in 62% of *SPG11* cases, one of the most frequent AR-HSPs, and up to 83% in *DDHD2*, a rare, severe HSP [34]. Dysarthria is also frequent in *CAPN1* patients, involving 42/61 (69%) of cases. *CAPN1* dysarthria results from the combination of pyramidal, bulbar and cerebellar involvement. Skeletal anomalies are reported in up to 16/37 (43%) of cases, and are therefore more frequent than deep sensory deficit (12/53, 23%). Brain and spine MRI may show atrophy, especially of cerebellum, brainstem and upper cervical cord (17/49 patients, or 35%, had abnormal MRI). Finally, the intra-familial and intra-variant phenotypic variability suggests individual factors modulating the variant consequences.

To reflect its clinical variability, the name ‘*CAPN1*-associated neurodegeneration’ has been proposed to describe the disease in patients carrying disease-causing variants in this gene [20]. It is now clear that some HSP genes are shared with many other neurodegenerative disorders [1]. However, screening in populations displaying early-onset Parkinson’s disease and youth-onset amyotrophic lateral sclerosis did not find any *CAPN1* pathogenic variants [16]. Notably, some of the *CAPN1* consanguineous cases also carry other pathogenic variants in *DYSF* or *MEFV*, so the phenotype in these cases may result from a synergistic effect of these two genes [28, 33]. The patient with *CAPN1* and *DYSF* pathogenic variants did indeed have a severe muscle weakness attributed to the muscular dystrophy.

In contrast to previous reports, we point out the importance of psychological assessment, with 7/17 cases (41%) described here being prone to depression. Depression is rarely reported and probably rarely assessed in spastic ataxia except for *SCA1* and *SCA3* [35]. We think that mood disorders are underestimated in HSPs; indeed, a previous study found that a half of HSP patients were subject to depression [36]. Whether this is a consequence or part of the pathological process is unclear as most published series lack this clinical information. In addition, cognitive impairment seems to occur during the evolution of the disease after two decades, as we have seen in two of our oldest cases. Such features have previously been reported only once, after 23 years of evolution and in the form of a frontotemporal dementia-like presentation associated with the same variant as one of our cases (c. 1176G>A) [25].

*CAPN1* causative variants result especially in deficiency of calpain-1-mediated cleavage of PH domain and leucine rich repeat protein phosphatase 1 (PHLPP1) [4]. This deficiency is suggested to cause inhibition of the Akt pro-survival pathway and, then, neurodegeneration. Activation of the Akt-pathway or inhibition of PHLPP1 is a therapeutic approach effective in mice to prevent neurodegeneration caused by *CAPN1* disease-causing variants [4]. This may open avenues of therapeutic intervention in the future.

## Conclusion

We described a large series of 21 patients from 13 families with *CAPN1* pathogenic variants causing various degrees of spastic ataxia, including nine novel variants.

*CAPN1* disease-causing variants are a rare cause of spastic ataxia of young adult onset, often involving consanguineous families. Truncating variants are the most frequent gene alterations. Interestingly, we found with the analysis of all the previously reported cases and our cases that the non-truncating missense variants are associated with an earlier age at onset than the truncating ones, which argues in favour of a stronger deleterious effect of the mutant protein compared with the truncating forms, which are probably degraded through non-sense-mediated mRNA decay. The associated clinical presentation is variable. Dysarthria and spasticity are the core features with mild cerebellar signs. Other signs are rarer, but some cases with cognitive impairment and/or depression indicate that these signs should be assessed specifically in the follow-up of spastic ataxia with *CAPN1* causative variants.

Many cases remain to be diagnosed and more data should be collected to know more about the natural history of this form.

## Supplementary information

Supplementary Table 1 Full clinical table of our new *CAPN1*-mutated cases (PDF 625 kb)

Supplementary Table 2 Literature review of all published *CAPN1*-mutated cases (until 10/2020) (PDF 785 kb)

## Data Availability

All CAPN1 variants identified in this study have been declared to the ClinVar database (http://www.ncbi.nlm.nih.gov/clinvar).
