# Supplementary material for: Increasing involvement of CAPN1 variants in spastic ataxias and phenotype-genotype correlations
Source: Neurogenetics. 2021 Jan 23;22(1):71–9. doi: 10.1007/s10048-020-00633-2 (PMC7997841; doi:10.1007/s10048-020-00633-2)
Supplement: Supplementary file 1 — Full clinical table of our new CAPN1-mutated cases (PDF 625 kb) [file 10048_2020_633_MOESM1_ESM.pdf]

Family		FSP-479	FSP-794		FSP-327			K4	K3	11264-SA	FSP-407	SR37-12333		SAL-620		5262	FSP-1225				K1		K2
Individual		**4**	7	**8**	770	**771**	778	**II:1**	**II:2**		**14**	**1**	3	5	**8**	**1**	7	**8**		12	**II:8**	II:7	**II:6**
Gender		M	M	M	M	F	M	M	M	M	M	F	F	M	F	F	M	F		F	M	F	F
Origin		France	Portugal		Algeria			Czech Republic	Sweden	Italy	Portugal	Portugal		France		France	Egypt				Iraq (Kurdish)		Iraq
Variant 1		**c.254G>A (p.Trp85*)**	c.618_619del (p.Gly208Glnfs*7)		**c.623G>A (p.Gly208Asp)**			**c.1005G>A (p.Trp335*)**	**c.1129_1133del (p.Arg377Glufs*25)**	c.1153C>T (p.Arg385*)	c.1176G>A (p.Trp392*)	c.1176G>A (p.Trp392*)		c.1176G>A (p.Trp392*)		c.1605+5G>A (p.E523Kfs*28)	**c.1697dup (p.Leu566Phefs*7)**				**c.1969G>T (p.Glu657*)**		**c.1969G>T (p.Glu657*)**
Variant 2			**c.1341G>C (p.?) [SPLICE]**					c.1605+5G>A (p.E523Kfs*28)	**c.1165+1G>A (p.?)**					**c.1418_1419del (p.Arg473Leufs*53)**									
Homozygous/Consanguinity		+/+	-/-		+/+			-/-	-/-	+/-	+/+	+/+		-/-		+/-	+/+				+/-		+/-
Age at onset (years)		16	34	31	19	24	6	30	22	14	15	28	30	28	38	42	18	23		36	20	28	24
Age at exam (years)		24	38	37	25	32	21	50	31	43	27	60	55	58	48	43	36	35	41	45	44	48	54
Severity at exam or at age (years) indicated		2	1	1	3	3 at 25 ; 4 at 32	2	3	3	3 at 31 and 4 at 44	1 at 22 ; 3 at 32	5 at 60; died at 63	4 at 55; 6 at 68	5	3	3	4	3	4	5	3	5	5
Pyramidal signs	LL spasticity	+	+	+	+	+	+	+	+	+	+	+	+	+	+	+	+	+	+	+	+	+	+
	LL hyperreflexia	+	na	+	+	+	+	+	+	+	+	+	+	+	+	+	na	+	+	na	+	+	+
	UL spasticity	+	na	na	+	+	+	-	-	+	-	+	+	+	+	+	na	-	-	-	-	-	-
	UL hyperreflexia	+	na	+	+	+	+	-	-	+	+	+	+	+	+	+	na	+	+	na	-	+	+
	Extensor plantar reflex	+	na	+	+	+	+	+	+	+	+	+	+	+	+	+	na	+	+	na	+	+	+
	Sphincter dysfunction	+ (urinary urgency)	-	na	-	-	-	-	-	-	+ (urinary urgencies)	+	+	+	+ (mild urinary urgency)	+	na	+ (and anal incontinence)	+ (and anal incontinence)	na	-	+ (severe)	+
LL weakness		+ (proximal and distal)	-	-	+ (proximal and distal)	+ (proximal and distal)	+ (proximal and distal)	+ (proximal and distal)	-	+ (distal)	-	+	+	+ (proximal)	+ (proximal)	+	na	+ (distal)	+ (distal)	+	+ (proximal and distal)	+ (proximal and distal)	+ (proximal and distal)
Amyotrophy		-	na	-	-	-	-	-	-	na	-	na	na	-	-	na	na	-	-	na	na	na	na
Cerebellar ataxia		+	+	-	+	+	+	-	-	+	-	-	-	+	+	+	na	- (left UL tremor when finger nose test)	+	na	-	-	- (mild dysmetria in the UL)
Ocular movement disorder		-	+ (saccadic pursuit)	na	-	-	-	-	-	-	-	-	-	-	-	-	na	-	-	na	-	+ (hypometric saccades)	+ (broken eye pursuit)
Dysarthria		+ (slow, spastic)	-	-	+ (cerebellar)	+ (cerebellar)	+ (cerebellar)	-	-	+ (spastic)	-	-	+ (slow, spastic)	+ (velar insufficiency)	+ (cerebellar)	-	na	-	+ (spastic)	+	-	+ (bulbar)	+ (bulbar)
Dysphagia		-	-	-	-	-	-	-	-	-	-	-	-	+	+	-	na	-	-	na	-	+ (severe)	+ (mild, no need to modify diet)
Sensory deficit		-	+ (deep sensory)	+ (deep sensory)	-	-	-	-	-	+ (deep + superficial sensory)	-	+ (deep sensory)	+ (deep sensory)	+ (deep sensory)	+ (deep sensory)	-	na	-	-	na	-	-	-
Peripheral neuropathy ENMG		na	na	na	na	+ (peripheral neurogenic)	na	-	-	-	-	na	na	-	-	-	na	na	na	na	-	-	-
Skeletal anomalies		+ (scoliosis, hollow left foot and C2-3 fusion with supernumerary hemivertebra)	+ (scoliosis and pes cavus)	+ (pes cavus)	-	-	-	-	-	-	-	-	-	+ (pes varus)	+ (mild scoliosis and pes cavus)	-	na	+ (pes cavus and dental abnormalities)		na	-	-	-
Extrapyramidal signs		-	-	-	-	-	-	-	-	-	-	-	-	-	-	-	na	-	+ (orofacial dystonia)	na	-	-	-
Intellectual involvement		-	-	-	-	-	-		- (MoCA 30)	-	-	+	+	-	-	-	na	-	-	na	- (MoCA 27)	- (MoCA 28)	- (MoCA 25)
Psychiatric features		-	na	na	-	-	-	-	+ (history of depression)	-	+ (bipolar disorder)	-	-	+ (depression)	+ (depression)	-	na	+ (anxiety and depression)	+ (anxiety and depression)	na	-	+ (PTSD, recurrent depression)	+ (depression)
Imaging	Brain MRI	N	na	na	na	na	A (cerebellar vermian atrophy)	N	N	N	A (pyramidal tract abnormalities)	N	N	A	N	A (4 aspecific white matter FLAIR hyperintensities with 1 in cerebellum)	na	N		na	N	N	N
	Spine MRI	na	na	na	na	na	na	N	N	na	N	na	na	na	N	N	na	A (dorsal spinal cord atrophy)		na	N	N	N
Comorbidities and other observations		Post-infectious deafness				muscle biopsy without mitochondrial abnormality		Disc herniation L5	History of depression						HTA, uterine fibroid			Bilateral cataract	Bilateral cataract	Bilateral cataract at 43 years		Asthma, frozen shoulder	

Supplementary Table 1. Full clinical table of our new *CAPN1* mutated cases.
