# Supplementary material for: Increasing involvement of CAPN1 variants in spastic ataxias and phenotype-genotype correlations
Source: Neurogenetics. 2021 Jan 23;22(1):71–9. doi: 10.1007/s10048-020-00633-2 (PMC7997841; doi:10.1007/s10048-020-00633-2)
Supplement: Supplementary file 2 — Literature review of all published CAPN1-mutated cases (until 10/2020) (PDF 785 kb) [file 10048_2020_633_MOESM2_ESM.pdf]

Variants	Ref.	Fam Nb	Patient Nb	Truncating	Fam	Zygosity	Cons	Case	Gender	Origin	Onset age (year)	Age at exam	Severity *	LL spasticity	LL hyperreflexia	UL spasticity	UL hyperreflexia	Babinski	Cerebellar ataxia	Dysarthria	Type of dysarthria	Skeletal anomalies	LL Weakness	Amyotrophy	Ocular movement disorder	Eye fundus	Sphincter dysfunction	Dysphagia	Sensory deficit	Peripheral neuropathy (EMG)	EMG comment	Extrapyramidal signs	Intellectual involvement	Epilepsy or psychiatric features	Brain MRI	Spine MRI	Other	Pure HSP
c.142C>T (p.Arg48*)	Peng, 2019	1	1	Y	A	Hom	Y	3	M	China	18	38	2	Y	Y		Y	Y	N	N		N		N	N		N		N	N			N		N	N	muscle hypertonic in LL, bilateral positive Hoffmann's reflex	Y
c.188dup (p.Val64Glyfs)	Wang, 2016	1	1	Y	SAL-584	Hom	Y	SAL-584-005	F	Italy	25	52	6	Y	Y		Y	Y	Y	Y	cerebellar				N		Y	Y	N	N					N			N
c.188dup (p.Val64Glyfs)	Lai, 2020	1	1	Y	1	Hom	N	II-1	M	China	27	41	3	Y	Y	Y	Y	Y	Y	N			Y		N		N		N							N		N
c.188dup (p.Val64Glyfs)	Lai, 2020	1	2	Y	4	Hom	Y	IV-1	M	China	21	41	3	Y	Y	Y	Y	Y	Y	Y	slurred speech				N		N		N							N		N
				Y	4	Hom	Y	IV-2	F	China	21	40	3	Y	Y	Y	Y	Y	Y	Y	slurred speech				N		N		N							N		N
c.221G>A (p.Gly74Asp) + c.1418G>T (p.Arg473Leu) + c.911C>T (p.Thr304Met)	Travaglini, 2017	1	1	N		Het	N		M	Italy	1	16	2	Y	Y			Y	N														N		N	N		Y
c.337+1G>A (p.Leu112ins9)	Wang, 2016	1	2	N	R	Hom	Y	1	F	Bangladesh	16	47	6	Y	Y				Y	Y	bulbar							Y	N	N			Y		A	A		N
				N		Hom	Y	2	F	Bangladesh				Y	Y				Y																			N
c.338-1G>A (p.Asp114Thrfs*62)	Xia, 2020	1	3	Y		Hom	Y	II.3	M	China	30	48	5	Y	Y	Y	Y	Y	Y	Y					N			Y	N				N		A	A	ankle clonus	N
				Y		Hom	Y	II.6	F	China	30	45	5	Y	Y	Y	Y	Y	Y	Y					N			Y	N				N				died of gastrointestinal cancer	N
				Y		Hom	Y	II.9	F	China	29	44	5	Y	Y	Y	Y	Y	Y	Y					N			Y	N				N					N
c.397C>T (p.Arg133*)	Shetty, 2018		1	Y	10	Hom	Y	2	M	Turkey	23	42	5	Y	Y				N	Y	very mild	Y (pes planus)	Y						N						N	A		Y
c.397C>T p.Arg133*) + DYSF: c.3113G>A (p.Arg1038Gln)	Shetty, 2018	1	1	Y		Hom	Y	3	F	Turkey	20	36	3	Y	Y	Y	Y	Y	N	Y	nasal speech		Y		N		Y		N	N					N	N	limb girdle muscular dystrophy, type 2B (LGMD 2B) with profound muscle weakness	Y
c.407del (p.Pro136Argfs*40) + c.1605+5G>A (p.E523Kfs*28)	Gan-Or, 2016	1	2	Y	C	Het	N	IV-7	M	USA (Idaho and Utah)	33	35	2	Y			Y	N	Y			Y			N	N	N								A	A	ankle clonus	N
				Y		Het	N	IV-13	F	USA (Idaho and Utah)	19	31	3	Y	Y		Y	Y	Y			Y	Y	N	N	N	Y		N						N		ankle clonus	N
c.463C>T (p.Gln155*) + c.1142C>T (p.Ala381Val)	Wang, 2016	1	1	Y	SAL-399-073	Het	Y	SAL-399-073	F	France and Spain	20	46	6	Y	Y		Y	Y	Y	Y	cerebellar, at onset	Y (scoliosis)	Y	Y	Y		Y	Y	Y (vibration sense)	Y	mild axonal sensoryperipheral neuropathy	Y (hypokinesia)			A		ankle clonus	N
c.614T>G (p.Leu205Arg) + c.1142C>T (p.Ala381Val)	Kim, 2019	1	1	N	F	Het	N	F	F	Korea	13	29	6	Y	Y			Y	Y	Y	cerebellar, onset sign		Y	Y	N	N	N		Y (vibration sense)			Y (postural tremor)	N		A	A	ankle clonus	N
c.675C>A (p.Tyr225*)	Melo, 2018	1	2	Y	C	Hom	Y	1	F	Brazil	20	46		Y	Y				Y																			N
				Y		Hom	Y	4	M	Brazil	35	51		Y	Y				Y																			N
c.759+1G>A (p.Ser198Valfs*5)	Tadic, 2017		2	Y		Hom	Y	1	F	Germany	29	39	3	Y	Y	y	y	y	Y	Y	mild	Y (pes varus)			Y										A	N		N
c.759+1G>A (p.Ser198Valfs*5) + MEFV: p.M680I and p.V726A	Tadic, 2017			Y		Hom	Y	2	F	Germany	33	37	2	Y	Y			Y	Y																		AR familial Mediterranean fever (FMF)	N
c.759+1G>A (p.Ser198Valfs*5)	Wei, 2019	3	2	Y	6	Hom	Y	Case 6 = IV.3	M	China	20	34		Y	Y		Y	Y	N	Y			N					Y	N								ankle clonus	Y
				Y		Hom	Y	IV.2	F	China				Y	Y		Y	Y	N																			Y
c.759+1G>A (p.Ser198Valfs*5)	Lai, 2020		2	Y	3	Hom	N	II-1	F	China	33	44	2	Y	Y	N	Y	Y	Y	N					N		N		N									N
				Y	3	Hom	N	II-2	M	China	38	42	2	Y	Y	N	Y	N	Y	N					N		N		N							A		N
c.843+1G>C (p.?)	Shetty, 2018	1	1	Y	11	Hom	Y	4	M	Punjab (India)	37	42	3	Y	Y	Y	Y	Y	Y				Y		N				Y (vibration sense)						N	N		N
c.853c>T (p.R285*)	Rahimi Bidgoli, 2020	1	2	Y		Hom	Y	2	F	Iran	25	37	3	Y	Y	Y	Y	Y	N	N		N	Y		N		N		N	N			N	N	N	A	hypothyroidism	Y
				Y		Hom	Y	3	M	Iran	25	31	3	Y	Y	Y	Y	Y	N	N		N	Y		N		N		N	N			N	N	N	N		Y
c.853C>T (p.R285*)	Lai, 2020	1	1	Y	5	Hom	Y	IV-4	M	China	28	46	2	Y	Y	N	Y	Y	N	N			Y		N		N		N							N		Y
c.884G>C (p.Arg295Pro)	Gan-Or, 2016	1	3	N	A	Hom	Y	V-1	M	Morocco			2																									Y
				N		Hom	Y	V-2	F	Morocco	20	31	2	Y	Y		Y	Y	N	Y		Y	Y	N			Y		N	N		N						Y
				N		Hom	Y	V-4	F	Morocco			2																									Y
c.959del (p.Tyr320Leufs*73)	Cotti Piccinelli, 2019	1	2	Y		Hom	Y	1	M	Italy	25	33		Y	Y			Y											N	N					N	N	congenital thyroid hemi-hypoplasia	Y
				Y		Hom	Y	2	F	Italy	30	42		Y	Y			Y											N	N					N	N	congenital thyroid hemi-hypoplasia	Y
c. 994G>A (p.Gly332Arg)	Kocoglu, 2018	1	1	N	1	Hom	Y	1	F	Turkey	21	37	4	Y	Y	Y	Y	Y	Y	Y			Y							N					N	N	Keratoconus	N
c.1015C>T (p.Arg339*)	Peng, 2019	1	1	Y	B	Hom	Y	1	F	China	36	41	3	Y	Y		Y	N	Y	N		N		Y	N		N		N				N		N	N	bilateral positive Hoffmann's reflex	N
c.1153C>T (p.Arg385*)	CLINVAR only by GeneDx (USA)		LINK	Y																																		Y
c. 1176G>A (p.Trp392*)	Kocoglu, 2018		2	Y	2	Hom	Y	1	F	Turkey	15	54	4	Y	Y	Y	Y	Y	Y	Y										N					N	N		N
				Y		Hom	Y	2	F	Turkey	15	45	4	Y	Y	N			Y	Y																		N
c.1176G>A (p.Trp392*)	Melo, 2018		3	Y	A	Hom	Y	1	F	Brazil				Y	Y				Y	Y																		N
				Y		Hom	Y	4	F	Brazil				Y	Y				Y	Y																		N
				Y		Hom	Y	6	M	Brazil				Y	Y				N	Y																		Y
			1	Y	B	Hom	Y	1	F	Brazil	22	37		Y	Y				N																			Y
		5	1	Y	D	Hom	Y	5	M	Brazil	38	38		Y	Y				Y																			N
c. 1176G>A (p.Trp392*)	Souza, 2019		1	Y		Hom	Y		F	Brazil	25	48	6	Y	Y	Y	Y	Y				Y (scoliosis and pes cavus)	Y				Y		Y	Y	moderate chronic axonal sensorimotor polyneuropathy of LL		Y	Y (chronic depression, suicidal ideation and psychotic symptoms started at 45 (auditory and visual hallucinations)	A	N	frontal release signs, language, visuospatial and executive skills are affected, ankle clonus	N
c.1176G>A (p.Trp392*) + c.618_619delAG (p.Gly208Glnfs*7)	Melo, 2018		1	Y	D	Het	Y	1	F	Brazil	30	42		Y	Y				Y															Y (chronic depression)				N
c.1271T>G (p.Met424Arg) + c.1943-5_1943del	Kim, 2019	1	1	Y+N	E	Het	N	E	M	Korea	19	27	2	Y	Y			Y	Y	Y	cerebellar and dysphonia		Y	N	Y	N	N		N	N		N			A		ankle clonus	N

(p.?)																																				
c.1353+2T>C (p.?) + c.1729+1G>A	GarciaBerlanga, 2019	1	1	Y		Het			F	Argentina	23	38	3	Y	Y	Y	Y	Y	Y	Y					Y							Y (mild cervical dystonia with bradykinesia)	N	N		N
c.1442G>A (p.Arg481Gln) + c.1493C>T (p.Pro498Leu)	Lai, 2020	1	1	N	6	Het	N	II-1	F	China	19	23	2	Y	Y	N	Y	Y	N	N					N		N		N						N	Y
c.1493C>T (p.Pro498Leu) + c.1852C>T (p.Arg618Trp)	Lai, 2020	1	1	N	2	Het	N	II-1	M	China	33	42	2	Y	Y	Y	Y	Y	N	N			Y		N		N		N						N	Y
c.1534C>T (p.Arg512Cys)	Wang, 2016		2	N	Tun-627	Hom	Y	Tun66273	F	Tunisia	23		2	Y	Y		Y	Y	Y	Y		N							N					A		N
		2		N		Hom	Y	Tun66275	F	Tunisia	20		2	Y	Y		Y	Y	Y	Y		N							N							N
c.1534C>T (p.Arg512Cys)	Lambe, 2018		1	N		Hom	N		F	Ireland (settled travelling community)	13	33	6	Y	Y	Y	Y	Y	Y	Y									N				N	A	N	N
c.1579C>T (p.Gln527*)	Gan-Or, 2016		5	Y	B	Hom	Y	IV-1	M	Morocco	35	47	2	Y	Y		Y	Y	N	Y	paralytic with akinetic face	N	Y	N	N	N	N		Y (all modes)	Y	moderate sensory axonal neuropathy predominating in LL					N
		1		Y		Hom	Y	IV-2	F	Morocco	36	44	3	Y	Y		Y	Y	Y	Y	paralytic with moderate facial hypokinesia	Y	Y	N	N	N	N			Y	moderate sensory axonal neuropathy predominating in LL		N			N
				Y		Hom	Y	IV-4	M	Morocco	22	42	2	Y	Y		Y	Y	N	Y		Y	Y	N	N	N	N							N	N	Y
				Y		Hom	Y	IV-5	M	Morocco	39	40	2	Y	Y		Y	N	Y	Y		N	N	N	Y	N	N									N
				Y		Hom	Y	IV-9	F	Morocco	24	30	2	Y	Y		Y	Y	N	Y		Y	N	N	N	N	N			N		N				Y
c.1605+5G>A (p.E523Kfs*28)	Hengel, 2020			Y		Hom	Y			Palestinian or Israeli Arab				Y	Y																					Y
		1	3	Y	AQ28	Hom	Y			Palestinian or Israeli Arab				Y	Y																					Y
				Y		Hom	Y			Palestinian or Israeli Arab				Y	Y																					Y
c.2118+1G>T (p.?)	Shetty, 2018	1	1	Y	9	Hom	Y	1	F	Japan	37	42	2	Y	Y	Y	Y	Y	Y	N			Y				N	Y (solid)	N					N	N	N

Supplementary Table 2. Literature review of all published *CAPN1* mutated cases (until 10/2020).

Cons= consanguinity, Fam= family, Het=heterozygous, Hom=homozygous, LL=lower limbs, N=no, UL=upper limbs, Y=yes

*: SPATAX-EUROSPA disability score
